# Comparative evaluation of anti-angiogenic effects of noscapine derivatives

**DOI:** 10.6026/97320630014236

**Published:** 2018-05-31

**Authors:** Rajesh K. Meher, Manas Ranjan Naik, Banajit Bastia, Pradeep K. Naik

**Affiliations:** 1 Department of Biotechnology & Bioinformatics, Sambalpur University, Jyoti Vihar - 768 019, Sambalpur, Odisha; 2 Department of Pharmacology, VSS Institute of Medical Science & Research, Burla, Sambalpur, Odisha; 3 Environmental Toxicology & Electron Microscope Lab, ICMR-National Institute of Pathology, Safdarjung Hospital Campus, New Delhi-110029, India

**Keywords:** Angiogenesis, noscapine, Cl-noscapine, Br-noscapine, folate-noscapine, TNP-470, paclitaxel

## Abstract

Angiogenesis, the formation of new capillaries from pre-existing vessels, is essential for tumor progression. Synthetic derivatives of anti-cancer compound, noscapine (an opium alkaloid) such as Cl-noscapine, Br-noscapine and Folate-noscapine along with two of the reference compounds, TNP-470 and paclitaxel were examined for anti-angiogenic activities by using human umbilical vein endothelial cells (HUVECs). The noscapine derivatives showed anti-angiogenic activity albeit at high concentration compared to the reference compounds. All the tested compounds inhibited angiogenesis in a dose-dependent manner; the drug concentration causing 50% inhibition of cell survival was 11.87 μM for Cl-noscapine, 6.9 μM for Br-noscapine and 6.79 μM for folate-noscapine. Besides, all the noscapine derivatives significantly inhibited cord formation (IC50 for Cl-noscapine is 50.76 μM, for Br-noscapine is 90.08 μM and for folate-noscapine is 18.44 μM) as well as migration and invasion (IC50 value of Cl-noscapine is 28.01 μM, for Br-noscapine is 19.78 μM and for folate-noscapine is 10.76 μM) of endothelial cells. Based on these results, we speculated that the inhibitory effects on human endothelial cell proliferation of noscapine derivatives might be important for anti-angiogenesis.

## Background

Angiogenesis is a pre-determining phenomenon for the survival and progression of a variety of solid tumors. Tumors remain dormant in the absence of angiogenic stimulus. Angiogenesis is controlled by a sensitive physiological switch, which is triggered either by proangiogenic factors or antiangiogenic factors. These angiogenic factors are diverse in their function and broadly distributed both in intracellular and extracellular environments. In normal tissues antiangiogenic factors are predominant over proangiogenic factors. The most prominent pro-angiogenic factors are VEGF (vascular endothelial growth factor), bFGF (basic fibroblast growth factor), MMPs (Matrix metalloproteinases) and cyclo-oxygenase - 2 (COX-2). Inhibiting angiogenesis requires treatment with anti-angiogenic factors, or drugs that reduce the production of pro-angiogenic factors, prevent them binding to their receptors or block their actions. Inhibition of the VEGF pathway has become the focus of angiogenesis research as approximately 60% of malignant tumors express high concentrations of VEGF. As an example, treatment with bevacizumab (a monoclonal antibody against VEGF) has resulted in improved survival in colorectal cancer patients [1, 2]. Other anti-angiogenic agents like angiostatin [3, 4], anti-VEGF antibody [5], receptor tyrosine kinase inhibitors [6, 7, 8, 9], endostatin [10], and VEGF Trap [11] have been demonstrated to enhance radiotherapy's effects [12, 13]. The tubulin binding agent, taxanes, which is clinically used for the treatment of various types of cancer has reported to have antiangiogenic effect [14]. Taxanes interfere with the endothelial cell proliferation, migration and differentiation into capillary-like tubes to supply to a growing tumor [15, 16, 17]. However, the taxanes are plugged with severe side effects such as peripheral neuropathy, myelosuppression, alopecia, gastrointestinal toxicity, immunosuppression, cardiotoxicity, etc. Efforts to develop novel tubulin binding agents with improved toxicity profiles have resulted in a novel microtubule binding agent, noscapine, which has shown promise in this regard in both animal and human studies [18, 19, 20]. Recently it was shown that noscapine has anti-angiogenic activity similar to taxanes [21]. Noscapine downregulated hypoxia-mediated HIF-1α expression in human glioma cells, concomitantly with reduced secretion of the potent angiogenic cytokine, VEGF [21]. In addition, noscapine inhibited tubule formation by human umbilical vein endothelial cells (HUVECs) at high concentration. Based on these observations, and given its unique low toxicity profile, we hypothesized that noscapine derivatives might be the promising candidates to effectively inhibit tumor induced angiogenesis.

## Methodology

We have adopted 3 in vitro assays (developed by NCI) for assessing the anti-angiogenic activity of the noscapine derivatives, Cl-noscapine, Br-noscapine and folate-noscapine (synthetic derivatives) of natural compound, noscapine which is an opium alkaloid, less toxic, tubulin binding, anti-cancer agents [22]. Previously these derivatives of noscapine were tested as potent tubulin binding, less toxic, anti-cancer compounds against the NCI 60 cell lines panel [23, 24]. TNP-470 (NSC 642492) and paclitaxel (NSC 125973) are used as reference compounds in the assays.

### Growth inhibition assay

HUVEC (1.5 x 103) were plated in a 96-well plate in 100 μl of EBM-2 (Clonetic # CC3162). After 24h (day 0), the test compounds (100 μl) were added to each well at 2X the desired concentration (5-7 concentration levels) in EBM-2 medium. On day 0, one plate was stained with 0.5% crystal violet in 20% methanol for 10 minutes, rinsed with water, and air-dried. The remaining plates were incubated for 72h at 37 °C. After 72h, plates were stained with 0.5% crystal violet in 20% methanol, rinsed with water and air-dried. The stain was eluted with 1:1 solution of ethanol: 0.1M sodium citrate (including day 0 plate), and absorbance was measured at 540 nm with an ELISA reader. Day 0 absorbance was subtracted from the 72h plates and data were plotted as percentage of control proliferation (vehicle treated cells). IC50 (drug concentration causing 50% inhibition) was calculated from the plotted data.

### Cord formation assay

Matrigel (60 μl of 10 mg/ml) was placed in each well of an icecold 96-well plate. The plate was allowed to sit at room temperature for 15 minutes and then incubated at 37°C for 30 minutes to permit the matrigel to polymerize. In the mean time, HUVEC were prepared in EGM-2 (Clonetic # CC3162) at a concentration of 2 X 105 cells/ml. The test compounds were prepared at 2X the desired concentration (5 concentration levels) in the same medium. Cells (500 μl) and 2X drug (500 μl) mixed and 200 μl of this suspension were placed in duplicate on the polymerized matrigel. After 24h incubation, triplicate pictures were taken for each concentration using a Bioquant Image Analysis system. Drug effect (IC50) was assessed compared to untreated controls by measuring the length of cords formed and number of junctions.

### Cell migration assay

Cell migration was assessed using the 48-well Boyden chamber and 8 μm pore size collagen-coated (10 μg/ml rat tail collagen) polycarbonate filters (Osmonics, Inc.). The bottom chamber wells received 27-29 μl of DMEM medium alone (baseline) or medium containing chemo-attractant (bFGF, VEGF or Swiss 3T3 cell conditioned medium). The top chambers received 45 μl of HUVEC cell suspension (1 X 106 cells/ml) prepared in DMEM+1% BSA with or without test compound. After 5h incubation at 37°C, the membrane rinsed in PBS, fixed and stained in Diff-Quick solutions. The filter placed on a glass slide with the migrated cells facing down and cells on top removed using a Kimwipe. The testing performed in 4-6 replicates and five fields counted from each well. Negative un-stimulated control values subtracted from stimulated control and drug treated values and data plotted as mean migrated cell ± standard deviation. IC50 calculated from the plotted data.

## Results & Discussion

**Figure 1:**
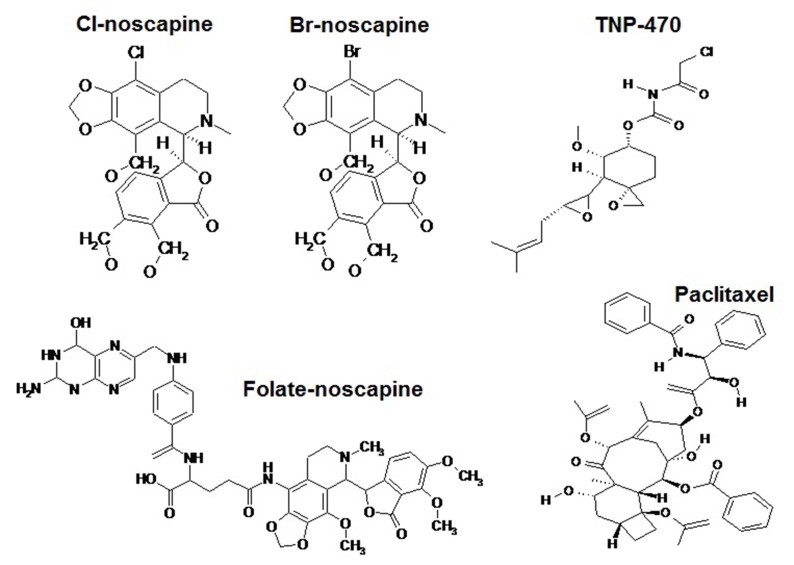
Molecular structure of Cl-noscapine, Br-noscapine, Folate-noscapine, TNP-470 and Paclitaxel.

**Figure 2:**
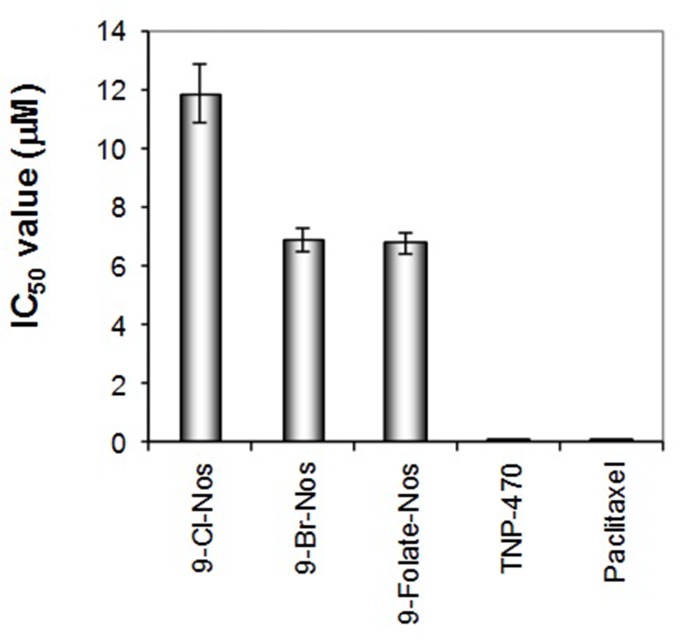
Growth inhibition of human endothelial cells by noscapinoids: 9-Cl-noscapine, 9-Br-noscapine and 9-Folate-noscapine. TNP- 470 and Paclitaxel were used as reference compounds.

**Figure 3:**
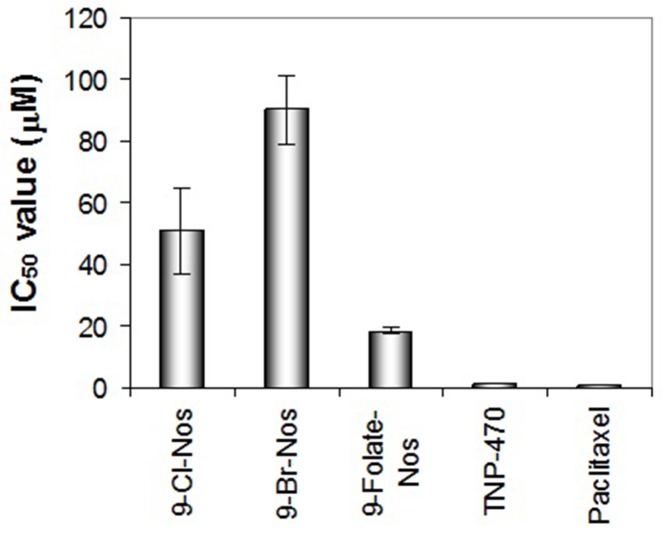
Inhibition of cord formation of human endothelial cells by noscapinoids: 9-Cl-noscapine, 9-Br-noscapine and 9-Folatenoscapine. TNP-470 and Paclitaxel were used as reference compounds.

**Figure 4:**
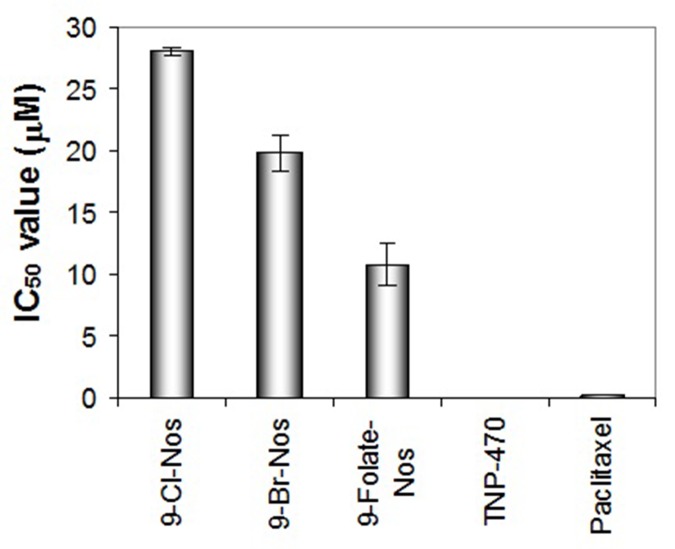
Inhibition of chemotaxis factor of human endothelial cells by noscapinoids: 9-Cl-noscapine, 9-Br-noscapine and 9-Folatenoscapine. TNP-470 and paclitaxel were used as reference compounds.

In order to test if the anti-angiogenic activity observed was due to direct cytotoxic effect on endothelial cells or due to inhibition of other angiogenesis cascade mechanisms, the cytotoxicity of noscapine derivatives: Cl-noscapine, Br-noscapine, folatenoscapine and two of the reference compounds, TNP40 and paclitaxel (Figure 1) was tested against HUVECs. The antiproliferative effects are shown in Figure 2. The noscapine derivatives showed a dose dependent anti-proliferative activity after 72 h of treatment compared to negative control in this study. IC50 was deduced from the logarithm regression equations obtained by plotting % cell viability versus concentration. All the tested compounds showed significant anti-proliferative activity (IC50 < 20 μM) to HUVECs; according to the National Cancer Institute (NCI) compounds with IC50 > 20 μM are not considered cytotoxic [25]. The drug concentration causing 50% inhibition of cell survival was 11.87 μM for Cl-noscapine, 6.9 μM for Brnoscapine and 6.79 μM for folate-noscapine. In order to illustrate the anti-angiogenic potential of noscapine derivatives in vivo, the extracts were tested on human endothelial cells as an in vivo angiogenesis model [26]. The results showed that noscapine derivatives significantly inhibited the new blood vessels formation and distorted the existing vasculature (Figure 3). All the noscapine derivatives significantly inhibited cord formation of endothelial cells (IC50 for Cl-noscapine is 50.76 μM, for Brnoscapine is 90.08 μM and for folate-noscapine is 18.44 μM). This result further supports the anti-angiogenic activity observed in vitro. The construction of a vascular network requires different sequential steps reproduction and wound healing. Under these conditions, neo-vascularization is tightly regulated. Unregulated angiogenesis may lead to several angiogenic diseases and is thought to be indispensable for solid tumor including the release of proteases from "activated" endothelial cells with subsequent degradation of the basement membrane surrounding the existing vessel, migration of endothelial cells into the interstitial space, endothelial cell proliferation, and differentiation into mature blood vessels. These processes are mediated by a wide range of angiogenic inducers, including growth factors, chemokines, angiogenic enzymes, endothelial specific receptors, and adhesion molecules. Thus, angiogenesis requires many interactions that must be tightly regulated in a spatial and temporal manner. Each of these processes presents possible targets for therapeutic intervention. Derivatives of noscapine were found to have significant inhibition of chemotaxis factor of human endothelial cells (Figure 4). The drug concentration that inhibits 50% of the chemotaxis of endothelial cells (IC50 value of Cl-noscapine is 28.01 μM, for Br-noscapine) is 19.78 μM and for folate-noscapine is 10.76 μM. Angiogenesis plays an important role in pathogenesis of various human diseases such as cancer, psoriasis, and diabetic retinopathy [27]. Therefore, noscapine derivatives, as a natural angiogenesis inhibitor, may provide new candidates for the treatment of these diseases. The selective and potent antiproliferative effect obtained against the cancerous cells, but not on normal cells, highlights noscapine derivatives as a potential source of new anti-cancer candidates.

## Conclusion

Taken together, noscapine derivatives (known as noscapinoids) showed robust anti-angiogenesis activity evident from the inhibition of growth of human endothelial cells, inhibition of cord formation of human endothelial cells and inhibition of chemotaxis factors responsible for the angiogenesis. Overall, the anti-angiogenesis activity of noscapine derivatives is not due to direct cytotoxicity on endothelial cells, but to inhibition of other vital steps in angiogenesis cascade, which needs further investigation.
